# Inhibition of STAT3 signaling as critical molecular event in HUC-MSCs suppressed Glioblastoma Cells

**DOI:** 10.7150/jca.77905

**Published:** 2023-02-27

**Authors:** Mingming Wang, Yufu Zhang, Min Liu, Yuna Jia, Jing He, Xiangrong Xu, Haiyan Shi, Yunqing Zhang, Jing Zhang, Yusi Liu

**Affiliations:** 1 Department of Cell Biology and Genetics, Medical College of Yan'an University, Yan'an 716000, Shaanxi Province, China; 2 Department of Hepatobiliary Surgery, Affiliated Hospital of Yan'an University, Yan'an 716000, Shaanxi Province, China; 3 Department of Pathology, Affiliated Hospital of Yan'an University, Yan'an 716000, Shaanxi Province, China; 4 Laboratory of Obstetrics and Gynecology, Affiliated Hospital of Yan'an University, Yan'an, 716000 Shaanxi Province, China

**Keywords:** human umbilical cord mesenchymal stem cell supernatant, glioblastoma, IL-6/JAK2/STAT3 signaling pathway

## Abstract

**Objective:** We investigated the effect of human umbilical cord mesenchymal stem cells (HUC-MSCs) supernatants on proliferation, migration, invasion, and apoptosis in glioblastoma (GBM) cell lines RG-2, U251, U87-MG, and LN-428, as well as their apoptosis and autophagy-mediated through IL-6/JAK2/STAT3 signaling pathway to explore the molecular mechanisms.

**Methods:** In this study, RG-2, U251, U87-MG, and LN-428 cells were treated with 9 mg/ml HUC-MSCs supernatants. Their responses to HUC-MSCs supernatants treatment and the status of STAT3 signaling were analyzed by multiple experimental approaches to elucidate the importance of HUC-MSCs supernatants for GBM.

**Results:** The results demonstrated that after treatment with HUC-MSCs supernatants, *in vitro* proliferation of RG-2, U251, U87-MG, and LN-428 cells were inhibited, and their sustained growth was also blocked. RG-2, U251, and U87-MG cells showed significant S phase accumulation, while LN-428 cells were blocked in G0/G1 phase. Their migratory invasive capacities were inhibited, and their apoptosis and autophagy ratios were increased. These effects were mediated through the IL-6/JAK2/STAT3 and its downstream signaling pathway.

**Conclusion:** Our data showed that HUC-MSCs supernatants had anti-tumor effects on GBM cells. It inhibited the proliferation, migration, and invasion of GBM cells and promoted their apoptosis. Negative regulation of the IL-6/JAK2/STAT3 signaling pathway enhanced apoptosis and autophagy in tumor cells, thereby improving the therapeutic effect on GBM.

## Introduction

Glioblastoma (GBM) is a primary brain tumor of the central nervous system in adults. It has been well-recognized that GBM was initiated by a subpopulation of cells termed glioblastoma stem cells (GSCs) 1, 2. Central nervous system (CNS) gliomas are classified into 4 grades according to the WHO classification. WHO grades 1-2 are classified as low-grade gliomas (LGG), whereas WHO grades 3-4 are classified as high-grade gliomas (HGG) 3, 4. The incidence of LGG is low, and the prognosis is usually good 5. GBM represents a grade 4 glioma. GBM is one of the most lethal and recurrent malignant solid tumors, accounting for 57% of all gliomas and 48% of primary CNS malignancies 6. The median survival of patients with GBM is only 14.6 months. Currently, conventional treatment includes neurosurgery, temozolomide (TMZ) dependent chemotherapy 1, 2, and chemotherapy. Although GBM cells rarely metastasize to other body organs, the treatment of GBM remains ineffective. The main reason for the poor prognosis of GBM is that conventional surgical resection often fails to eliminate the tumor, and the remaining tumor is often resistant to radiotherapy and chemotherapy, which inevitably leads to the recurrence of GBM 7. The main reason for this problem is that many anti-GBM drugs cause significant toxicity to the brain and/or body at high doses. Another bottleneck that makes treatment tricky is the obstruction of the blood-brain barrier (BBB), this structure is essential for protecting a healthy brain and preventing the transport of toxic substances in the blood 8-11, which also hinders progress toward effective treatment of GBM 12. However, it also impairs the ability of drugs to enter the brain for the effective treatment of lesions. It is the presence of these barriers that makes it difficult for many drugs to achieve the desired therapeutic efficacy. Therefore, there is an urgent need for a more effective strategy for treating GBM to complement or replace existing regimens.

Human umbilical cord mesenchymal stem cells (HUC-MSCs) possess multipotential differentiation abilities and self-renewal, and they are the most reliable source of stem cells in various cell therapies. Because of their multi-differentiation potential, high proliferation capacity, immune regulation, and self-replication 13-15, they are now regarded as seed cells for a wide range of applications in tissue engineering and biotherapy. HUC-MSCs have unique merits over bone marrow and adipose-derived MSCs in that they present no substantial ethical challenges, exhibit a low risk of viral transmission 16, have low immunogenicity, as well as being readily available and more primitive 17. HUC-MSCs have received more attention in the treatment of cancer. HUC-MSCs inhibit tumor growth by secreting relevant cytokines on their own. Studies have shown that HUC-MSCs can induce apoptosis and promote benign progression in esophageal, ovarian, and leukemia cancer cells 18-20. Furthermore, HUC-MSCs have stem cell characteristics, which means they can easily cross the blood-brain barrier and have tumor tropism 21. As a result, it can be used as a therapy to treat GBM. Although HUC-MSCs have been reported to be anti-GBM 21, there is a lack of exploration of extracting the supernatants of HUC-MSCs at different times to resist GBM.

Signal transducer and activator of transcription (STAT) proteins belong to a family of cytoplasmic transcription factors that can bind to DNA 22. The STAT family consists of seven structurally and functionally related proteins STAT1, STAT2, STAT3, STAT4, STAT5a, STAT5b, and STAT6 23. Of these, STAT3 is involved in a variety of biological processes, such as angiogenesis, cell proliferation, survival, and differentiation 24, 25. Upon tyrosine phosphorylation by receptor-associated tyrosine kinases, STAT3 translocates to the nucleus and activates the expression of downstream genes to regulate tumor cell growth, proliferation, differentiation, and metastasis 26. Previous studies have shown that the translocation and activation of STAT3 are affected by some factors that may enhance STAT3 activity, such as IL-6, Janus kinase 2 (JAK2), LIF, and EGF 27-29. In addition, Protein inhibitors of activated STAT3 (PIAS3) that bind to STAT3 have been identified 31. The binding of PIAS3 to STAT3 is only observed in cells that cause STAT3 activation. The DNA binding activity of STAT3 is blocked by PIAS3, thereby inhibiting STAT3-mediated downstream gene activation 30. PIAS3 is upregulated to inactivate STAT3, promoting apoptosis and autophagy in tumor cells. STAT3 plays a crucial role in multiple human cancers, especially in GBM, and it is in an over-activated state that is often associated with poor clinical prognosis 31. p-STAT3 was reported to affect the occurrence, development, and even prognosis of GBM 32, 33. However, whether HUC-MSCs supernatant mediates STAT3 inactivation in GBM cells remains unknown. We performed multiple experiments involving western blot, laser confocal, and Immunocytochemical Staining (ICC) to explore the mechanism of IL-6/JAK2/STAT3 signaling pathway regulation in GBM cells. And reported that C188-9 is a potent STAT3 inhibitor, which can target the phosphotyrosine (pY) peptide binding site in the STAT3 Src-homology (SH)2 domain 34, 35 to inhibit STAT3 activation. Based on this, novel therapeutic strategies targeting the IL-6/JAK2/STAT3 signaling pathway may open up new avenues for the regulation of long-term multilevel regulation of GBM cells.

## Materials and Methods

### Isolation, Culture, and Identification of HUC-MSCs

Umbilical cord tissues were obtained from healthy fetuses delivered by cesarean section at full term in the Obstetrics Department of Yan'an University Affiliated Hospital. With the approval of the Biomedical Ethics Committee of the Yan'an University School of Medicine, these umbilical cord tissues were obtained with the knowledge and permission of the mother and family. They were sent to Yan'an University Medical Experiment Center, where the umbilical cord tissue was isolated and cultured using the tissue block isolation method. The umbilical cord was repeatedly rinsed with saline to remove blood stains and cut into 5 sections, one umbilical vein and two umbilical arteries were carefully removed, and then cut into 2-3 mm sized tissue pieces and for inoculation in 75 cm^2^ culture flasks. The 75 cm^2^ culture flask inoculated with umbilical cord tissue blocks was inverted and placed in a 37°C, 5% CO_2_ cell culture incubator for 6 h, then 3-4 ml of HUC-MSCs complete medium (Procells Life Science Technology Co., Ltd.) was added. Cells could be seen crawling out from around the tissue block until about 7 days. The first passages could be made when the cells reached 80%-90% of the culture flask.

### Collection of HUC-MSCs Supernatants and Preparation of Lyophilized Powder

When P3-P8 generation HUC-MSCs grew to 80%-90% fused state, culture flasks full of HUC-MSCs were rinsed with PBS, and trypsin was added to each flask and digested for 2-3 min, then digestion was terminated with H-DMEM containing 10% FBS. The terminated cell suspension was collected and centrifuged at 1000 rpm for 5 min, discarded the supernatants and HUC-MSCs were harvested. The HUC-MSCs were inoculated according to 7×10^5^ cells/75cm^2^ cell culture flasks, and HUC-MSCs supernatants were collected in two approaches. In one approach, HUC-MSCs were incubated with 8-9 ml of H-DMEM containing 10% FBS for 24 h and replaced by 14 ml of H-DMEM without FBS for 24 h, 48 h, and 72 h respectively, and then HUC-MSCs supernatants were collected. After freezing and solidifying at -80°C, the frozen solidified supernatants were placed in a pre-cooled vacuum for evacuation, sublimated, and dried to prepare a lyophilized powder, weighed and dissolved in cell grade PBS and filtered through a 0.22 μm/ml filter. The filtered supernatants were collected in sterile centrifuge tubes and frozen at 4°C for short-term or -20°C for long-term storage. Alternatively, HUC-MSCs supernatants were collected using 8-9 ml of H-DMEM containing 10% FBS incubated for 24 h, 48 h, and 72 h replacement of 14 ml of H-DMEM without FBS for 24 h respectively, frozen solidified at -80°C, the frozen solidified supernatants were placed in a pre-cooled vacuum machine to evacuate, sublimated, and dried to prepare a lyophilized powder, weighed, dissolved in cells-grade PBS, filtered through a 0.22 μm/ml filter and the filtered supernatants were collected in sterile centrifuge tubes and stored frozen at 4°C for short-term storage or -20°C for long-term storage.

### GBM cells Culture and Cells Treatments

Human glioma cells U251, human brain astrocytoma cells U87-MG, and Human glioblastoma cells LN-428 were purchased from Otwo Biotech (Guangzhou, China). Rat glioma cells RG-2 cells were obtained from Ya Ji Biological (Shanghai, China). All cells were cultured in H-DMEM complete medium (GIBCO, Invitrogen) with a volume fraction of 10% fetal bovine serum (FBS, GIBCO, Invitrogen) and 1% penicillin-streptomycin (Solarbio, Beijing, China) at 37°C in a volume fraction of 5% CO_2_. RG-2, U251, U87-MG, and LN-428 cells were conditioned with 9 mg/ml of HUC-MSCs supernatants for 48 h. A total of 3×10^5^ cells were mounted on culture dishes (Nunc A/S, Roskilde, Denmark) and cultured for 24 h before further experiments. Dozens of cells-bearing coverslips were concurrently prepared under the same experimental condition using the high throughput coverslip-preparation dishes (Jet Biofile Tech. Inc., Guangzhou, China, China invention patent No. ZL200610047607.8), which were collected during drug treatments, fixed with cold acetone, and used for hematoxylin, and eosin (H&E) staining, and terminal deoxynucleotidyl transferase dUTP nick end labeling (TUNEL, Transgen, China) apoptosis assay.

### Cells Proliferation Assay

To determine the effect of GBM cells U251, U87-MG, LN-428, and RG-2 after incubation with HUC-MSCs supernatants or/and C188-9 (Alias: TTI-101, SperkJade, China). U251, U87-MG, LN-428, and RG-2 were inoculated on a 96-well plate and incubated with HUC-MSCs supernatants for 24 h, 48 h, and 72 h. Cell proliferation was analyzed by the Cells Counting Kit-8 assay (CCK-8, DOJINDO, Janan). The supernatants of HUC-MSCs were treated on U251, U87-MG, LN-428, and RG-2 cells for 48 h. Afterward, it was collected, resuspended with pre-cooled PBS, and then resuspended with 70% cold ethanol at -20°C overnight. The following day, cells were rinsed with pre-chilled PBS and stained using RNase and propidium iodide (PI) according to the manufacturer's instructions. Subsequently, the cell cycle was analyzed by NovoExpress (Agilent Technologies, USA).

### Annexin V/PI Staining Assay

The Annexin V-FITC Apoptosis Detection Kit (Nanjing Key Gen Biotech Co., Nanjing, China) was used for the quantification of apoptosis. 6×10^5^ cells per dish were seeded into 60 mm culture plates and collected after 48 h of HUC-MSCs supernatants treatment. Externalized phosphatidylserine was labeled with Annexin V-FITC conjugated for 15 min on ice. Propidium iodide (PI, 1 μg/mL) was added 10 min prior to FACS analysis. Active (annexin-/PI-), early (annexin+/PI-) and late apoptotic (annexin+/PI+) and necrotic cells (annexin-/PI+) were assigned. The labeled cells were identified by flow cytometry.

### Migration and Invasion Assays

Transwell chambers were assayed for cell migration invasion ability by applying 50 ul of Matrigel gel (1: 4 dilution) to the transwell chambers to be checked and incubated at 37°C for 30 min to form a gel. Approximately 3×10^4^ cells were inoculated onto the Matrigel gel-coated chambers and kept in an FBS-free cell culture medium with a concentration of 0 mg/ml or 9 mg/ml of HUC-MSCs supernatants or/and C188-9. H-DMEM containing 10% FBS was added to the lower chamber. The cells were incubated at 37°C and 5% CO_2_ for 24 h, fixed with 4% paraformaldehyde for 30 min, and stained with 0.1% crystalline violet for 20 min. Five fields of view were randomly selected for filming. Assessment of the migratory capacity of GBM cells was performed in the same steps as for the invasion assay, but without Matrigel.

### Immunocytochemical Staining/ICC

RG-2, U251, U87-MG, and LN-428 cells were conditioned with 9 mg/ml HUC-MSCs supernatants for 48 h. A total of 3 × 10^5^ cells were plated into culture dishes (Nunc A/S, Roskilde, Denmark) and cultured for 24 h before further experiments. Cells were collected after 48 h of drug treatment and fixed in 4% paraformaldehyde. Coverslips containing cells from each experimental group were subjected to ICC according to the SABC Immunohistochemical Staining Kit (Boster, California, USA). Briefly, Coverslips were washed with PBS (pH 7.4), incubated in 3% H_2_O_2_ for 10 min, and then incubated overnight at 4°C with the appropriate dilution of the first antibodies. The antibodies used were: IL-6 Polyclonal antibody (proteintech, 1: 1000), Rabbit Anti-JAK2 antibody (Bioss, 1: 500), STAT3 monoclonal antibody (ABclonal, China, 1: 200), phosphatidyl STAT3-Y705 rabbit pAb (ABclonal, China, 1: 200), PIAS3 (ABclonal, China, 1: 100), Survivin (ABclonal, China, 1: 100 ), VEGFA (ABclonal, China, 1: 100 ), Bcl-2 (proteintech, 1: 500), MCL-1 monoclonal antibody (ABclonal, China, 1: 100), c-Myc monoclonal antibody (Proteintech, 1: 100), Caspase-3 monoclonal antibody (Proteintech, 1: 500), Mouse Anti-Active Caspase 3 monoclonal ntibody (Bioss, 1: 500) Caspase 8 monoclonal antibody (Proteintech, 1: 500), MMP-2 monoclonal antibody (Proteintech, 1: 500), MMP-9 monoclonal antibody (Proteintech, 1: 500). Treatment with biotin-labeled goat anti-rabbit IgG for 20 min, followed by treatment with streptavidin-peroxidase complex for 20 min. Reactions were carried out using DAB chromogenic kit (DAB, Boster, California, USA). The staining results were evaluated by two independent researchers, depending on the intensity of the marker. If no immunolabelling was observed in the target cells, it was negative (-). Weakly positive (+) if the marker is faint. Moderately positive (++). Strongly positive (>++) if the marker is strong or significantly stronger than (++).

### Triple Immunofluorescent Labeling/IF

For triple immunofluorescence staining (IF), the coverslips with cells were rinsed by using phosphate-buffered saline (PBS, pH 7.4), fixed in 4% paraformaldehyde for 20 min, incubated in 0.1% Triton-X100 for 5 min, and closed with 3% BSA for 2 h. Primary antibodies LC3 ӀӀ/Ӏ (ABclonal, China, 1: 200) and Beclin-1 (Proteintech, Chicago, IL, USA, 1: 500) were mixed, and the coverslips were hatched overnight at 4°C overnight, coverslips were incubated with mouse anti-CoraLite488-conjugated goat anti-mouse IgG (H+L) and rabbit anti-Cy3-conjugated Affinipure goat anti-rabbit IgG (H+L) for 2 h. DAPI (Boster, California, USA) was incubated for 10 min to localize cell nuclei, mounted with an anti-fluorescence quenching sealing liquid (Solarbio, Beijing, China), observed and imaged in Laser Confocal Microscope (LSM800, ZEISS, Oberkochen, Germany).

### Western Blot Analysis

For Western Blot analyses, total cellular proteins were prepared from cells cultured under 9 mg/ml HUC-MSCs supernatants conditions. 50 µg/well or 30 µg/well of the sample proteins were separated by electrophoresis in 10% sodium doecylulfate--polyacrylamide gel electrophoresis (SDS-PAGE) and transferred to Millex-Millipore® 0.45 μm polypropylene difluoride membrane (Millipore®, MA, USA). The membranes were blocked with 10% skim milk in TBS-T (10 mM Tris-Cl, pH 8.0, 150 mM NaCl, and 0.5% Tween 20) at 37°C for 2 h, followed by incubation with the appropriate concentration of primary antibody overnight at 4°C. The antibodies used were: IL-6 Polyclonal antibody (proteintech, 1: 1000), Rabbit Anti-JAK2 antibody (Bioss, 1:500), STAT3 monoclonal antibody (ABclonal, China, 1: 1000), phosphatidyl STAT3-Y705 rabbit pAb (p-stat3, ABclonal, China, 1: 1000), PIAS3 (ABclonal, China, 1: 1000), Survivin (ABclonal, China, 1: 1000 ), VEGFA (ABclonal, China, 1: 1000 ), Bcl-2 (proteintech, 1: 1000), MCL-1 monoclonal antibody (ABclonal, China, 1: 1000), c-Myc monoclonal antibody (proteintech, 1: 1000), LC3 ӀӀ/ Ӏ (ABclonal, China , 1: 2000) and Beclin-1 (Proteintech, Chicago, IL, USA, 1: 2000), Caspase 3 monoclonal antibody (proteintech, 1: 1000), Mouse Anti-Active Caspase 3 monoclonal ntibody (Bioss, 1:1000), Caspase 8 monoclonal antibody (Proteintech, 1: 1000), MMP-2 monoclonal antibody (Proteintech, 1: 1000), MMP-9 monoclonal antibody (Proteintech, 1: 1000), Cyclin A2 (Proteintech, 1: 1000), CDK2 (Proteintech, 1: 1000), Cyclin D1 (Proteintech, 1: 1000) and CDK4 (Proteintech, 1: 1000). Subsequently, the membranes were incubated with HRP-conjugated anti-mouse or anti-rabbit IgG (Proteintech, Chicago, IL, USA) for 1.5 h. The bound antibodies were detected by enhanced chemiluminescence. Bound antibodies were detected using an enhanced chemiluminescence system (Syngene, India). The same experimental procedure was used for replating one by one with other antibodies until all parameters were checked.

### Statistical Analysis

Statistical analysis was performed by using Graphpad Prism 9.0 software (Graph-pad Software Inc., San Diego, CA). Two-way repeated measures ANOVA and two-tailed Student's t-test were used. *P* < 0.05 was considered statistically significant. The bar graphs present the mean ± standard deviation (SD) of the experimental data.

## Results

### HUC-MSCs Supernatants Collected by Different Methods Suppressed the Growth of GBM Cell Lines

**Figure 1:**
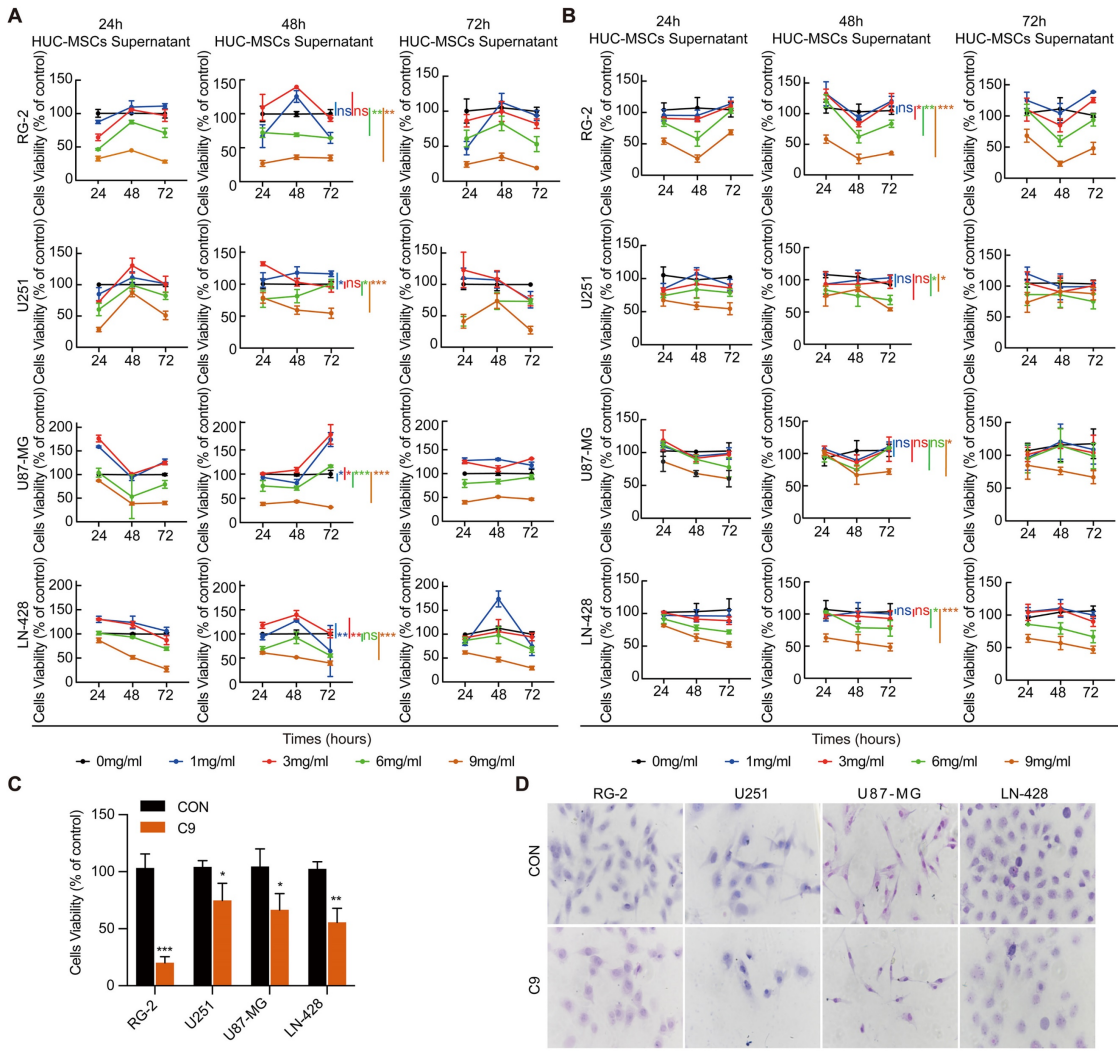
Growth inhibition of GBM cells by HUC-MSCs supernatant.** (A), (B)** and **(C)** CCK-8 assays: **(A)** HUC-MSCs were incubated with H-DMEM containing 10% FBS for 24 h and replaced by H-DMEM without FBS for 24 h, 48 h, and 72 h respectively, and then HUC-MSCs supernatants were collected. U251, U87-MG, LN-428, and RG-2 cells were treated with the collected HUC-MSCs supernatants for 24 h, 48 h, and 72 h, and then analyzed by the CCK-8 assay. **(B)** HUC-MSCs were incubated with H-DMEM containing 10% FBS for 24 h, 48 h, and 72 h, replaced by H-DMEM without FBS for 24 h. The HUC-MSCs supernatants were collected. U251, U87-MG, LN-428, and RG-2 cells were treated with the collected HUC-MSCs supernatants for 24 h, 48 h, and 72 h, respectively, and analyzed by the CCK-8 assay. **(C)** HUC-MSCs were incubated with H-DMEM containing 10% FBS for 48 h, replaced by H-DMEM without FBS, and continued for 24 h, then the collected supernatant of HUC-MSCs at 9 mg/ml was used as the effective concentration. **(D)** H&E morphological staining (inverted phase contrast microscope, × 40) was detected in GBM cells and treated for 48 h in the absence (CON) or presence (C9) of 9 mg/ml HUC-MSCs supernatants. HUC-MSCs supernatants, Human Umbilical Cord Mesenchymal Stem Cell Supernatants. ** P < 0.01, and *** *P* < 0.001 vs CON group; the error bars, the mean ± standard deviation.

We used two approaches to collect HUC-MSCs supernatants. One approach was to incubate HUC-MSCs with 8-9 ml of H-DMEM containing 10% FBS for 24 h and then with 14 ml of H-DMEM without FBS for 24 h, 48 h, and 72 h, respectively. Alternatively, HUC-MSCs supernatants were collected using 8-9 ml of H-DMEM containing 10% FBS incubated for 24 h, 48 h, and 72 h replacement of 14 ml of H-DMEM without FBS for 24 h, respectively. Subsequently, we assessed the effect of HUC-MSCs supernatants on the cell viability of U251, U87-MG, LN-428, and RG-2. We used two methods to collect HUC-MSCs supernatants that were divided into five different concentrations for the treatment of U251, U87-MG, LN-428, and RG-2 respectively for CCK-8 assay. The concentration of HUC-MSCs supernatants extracts and the treatment grouping of GBM cells were shown in Table 1. From the CCK-8 results, we observed that HUC-MSCs supernatants were maintained for 24 h, 48 h, and 72 h with varying sensitivity levels of U251, U87-MG, LN-428, and RG-2 to HUC-MSCs supernatants. U251, U87-MG, LN-428, and RG-2 showed concentration-dependent but not time-dependent inhibition. After the treatment of HUC-MSCs supernatants, we found that RG-2 cells were the most sensitive, followed by LN-428 and U87-MG, and U251 was the least sensitive (Fig. 1A and 1B). In particular, 9 mg/ml HUC-MSCs supernatants collected after 48 h incubation with H-DMEM containing 10% FBS replaced by H-DMEM without FBS for 24 h showed significant inhibition of the growth of U251, U87-MG, LN-428, and RG-2. For instance, after 48 h of treatment with 9 mg/ml HUC-MSCs supernatants, the cells viability of RG-2 was 19.96 ± 6.11% (*P* < 0.001), that of LN-428 was 63.93 ± 2.80% (*P* < 0.001). However, cell viability was 85.19 ± 2.90% (*P* < 0.01) and 78.55 ± 8.078% (*P* < 0.01) for U251 and U87-MG respectively. Overall, HUC-MSCs supernatants could selectively target GBM cells for toxic effects. Combining the results of the CCK-8 assay from both methods, we used H-DMEM containing 10% FBS for 48 h, then replaced H-DMEM without FBS to continue incubation for 24 h before collecting 9 mg/ml HUC-MSCs supernatants as the effective concentration for subsequent experiments (Fig. 1C).

To further determine the tumor-suppressive effect of U251, U87-MG, LN-428, and RG-2 by HUC-MSCs supernatants, we performed H&E morphological staining. The results showed that all GBM cells in normal culture were densely grown and morphologically plump, however, the morphological changes of the four cell types were different after treatment with 9 mg/ml of HUC-MSCs supernatants. RG-2 and LN-428 cells were partially crinkled and rounded, U87-MG cells were flattened and elongated, and U251 cells did not change significantly in morphology. At the same time, the number of cells in these four cell lines was reduced (Fig. 1D). In summary, HUC-MSCs supernatants inhibited the growth of GBM cells and influenced cell morphology.

### HUC-MSCs Supernatants Inducing GBM Cells to Arrest in G0/G1 or S Phase

**Figure 2:**
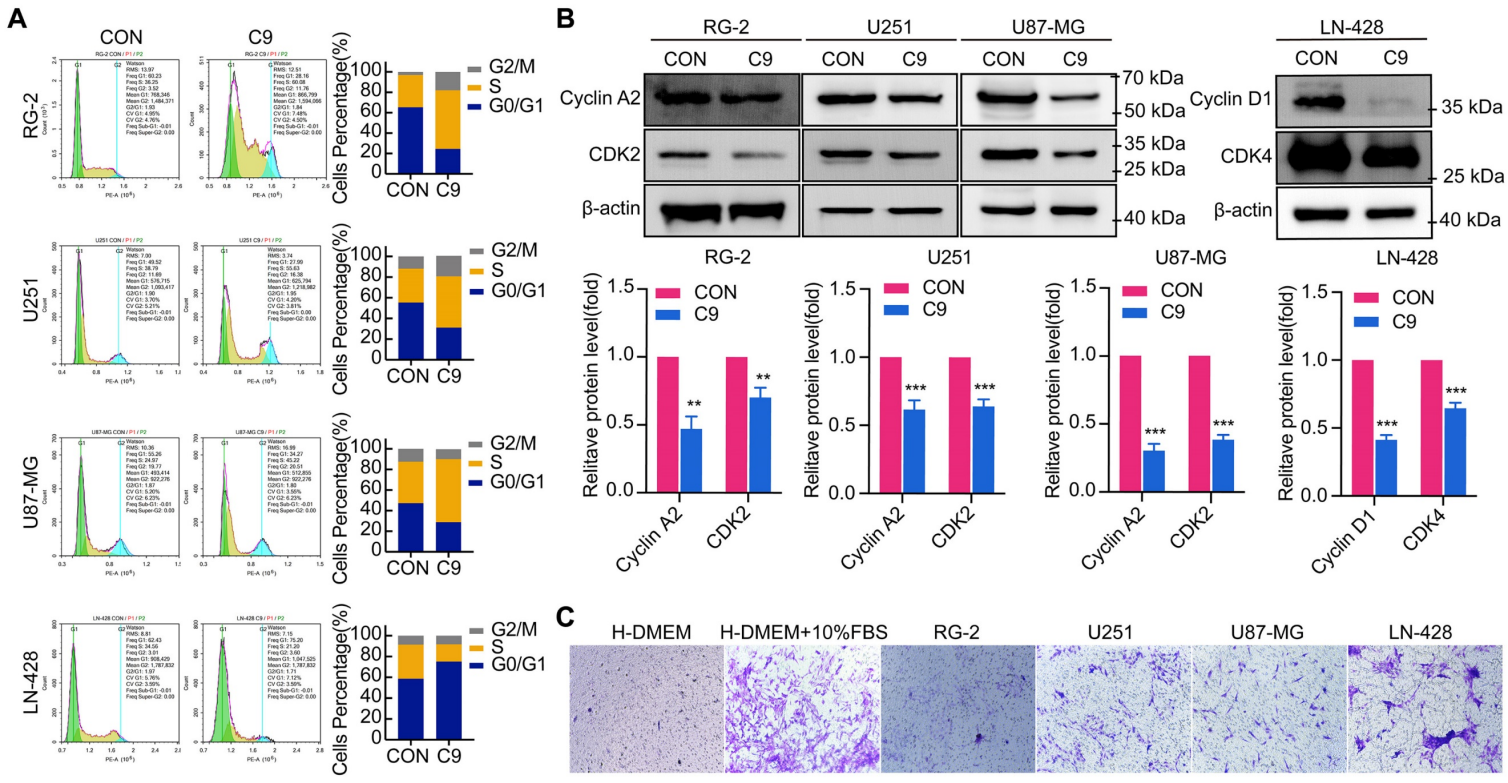
Cell cycle of U251, U87-MG, LN-428, and RG-2 cells was altered after treatment with 9 mg/ml HUC-MSCs supernatants for 48 h, and HUC-MSCs had tumor tropism. **(A)** Cell cycle distribution was detected with PI staining. **(B)** Western Blot. β-actin was used as a qualitative and quantitative control. **(C)** Transwell experiments confirmed *in vitro* that HUC-MSCs could migrate to GBM cells. CON, 0 mg/ml HUC-MSCs supernatant; C9, 9 mg/ml HUC-MSCs supernatants; *P < 0.05, **P < 0.01 and ***P < 0.001 vs CON group; the error bars, the mean ± standard deviation.

Cell cycle alterations are often influenced by cell proliferation. RG-2, U251, U87-MG, and LN-428 cell proliferation was inhibited by HUC-MSCs supernatants (Fig. 1C and D). To test whether HUC-MSCs supernatants affected the GBM cell cycle, we analyzed cell cycle changes by flow cytometry. The results showed that RG-2, U251, and U87-MG cells were stalled in the S phase by HUC-MSCs supernatants (Fig. 2A). In contrast, LN-428 cells were induced to stagnate in G0/G1 phase (Fig. 2A). We examined the expression levels of proteins associated with the corresponding cell cycle, such as Cyclin A2, CDK2, Cyclin D1, and CDK4, to further validate the effect of HUC-MSCs supernatants on the GBM cell cycle. Western Blot results suggested that the expressions of Cyclin A2, and CDK2 were reduced in RG-2, U251 and U87-MG treated with 9 mg/ml HUC-MSCs supernatants for 48 h. That is, Cyclin A2 expressions were reduced by 53%, 38%, and 70% in RG-2, U251, and U87-MG cells, and CDK2 expressions by 30%, 36%, and 62% respectively (Fig. 2B). The expressions of Cyclin D1 and CDK4 were reduced by 58%, and 35% in LN-428 cells treated with 9 mg/ml HUC-MSCs supernatants for 48 h (Fig. 2B). In summary, RG-2, U251 and U87-MG cells by HUC-MSCs supernatants were induced to block in S phase, whereas LN-428 cells were blocked in G0/G1 phase.

### HUC-MSCs Have the Ability to Tumor Tropism

The main challenge of anticancer drugs is poor tumor targeting 36. MSCs possess an inherent potential for tumor tropism and can serve as an ideal candidate 36. To test whether HUC-MSCs have tumor tropism *in-vitro*, we determined by the transwell assay that HUC-MSCs have tumor-specific tropism in RG-2, U251, U87-MG, and LN-428 cells (Fig. 2C), which provides a strong *in-vitro* experimental basis for targeted therapy of GBM.

### HUC-MSCs Supernatants Caused GBM Cells Apoptosis

**Figure 3:**
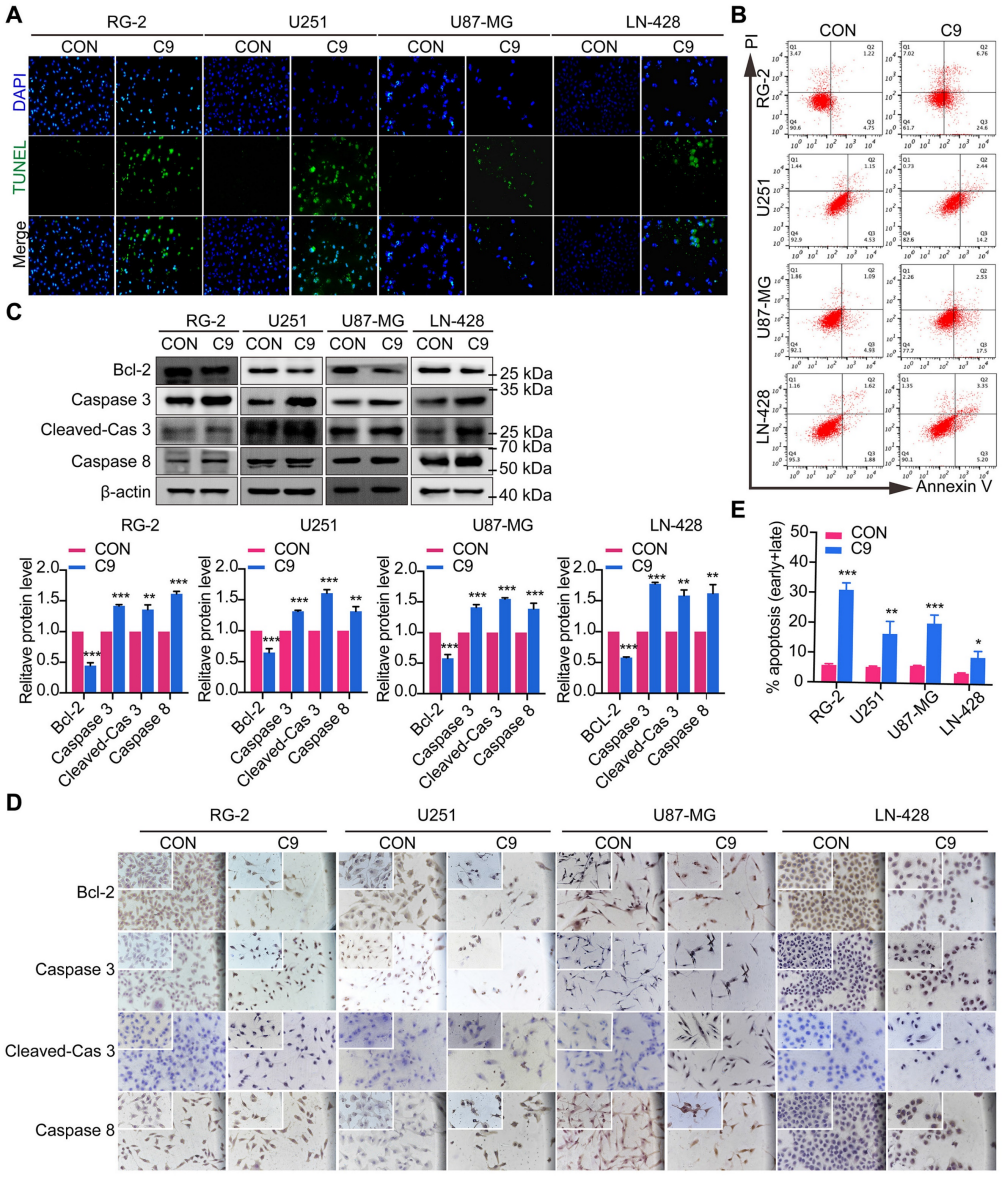
Apoptosis of U251, U87-MG, LN-428, and RG-2 cells after 48 h treatment with HUC-MSCs supernatants. **(A)** The images of the TUNEL apoptosis assay (× 20). **(B)** Flow cytometry analysis of Annexin V and PI in four kinds of GBM cell lines for apoptosis. **(C)** and** (D)** Expression of apoptosis-related proteins Bcl-2, Caspase 3, and Caspase 8. **(C)** Western Blot. β-actin was used as a qualitative and quantitative control. **(D)** ICC (× 20 and × 40). **(E)** Apoptosis of the four GBM cell lines tested by flow cytometry was statistically analyzed. CON, 0 mg/ml HUC-MSCs supernatant; C9, 9 mg/ml HUC-MSCs supernatants; TUNEL, Terminal Deoxynucleotidyl Transferase dUTP Nick End Labeling; ICC, Immunocytochemistry. *P < 0.05, **P < 0.01 and ***P < 0.001 vs CON group; the error bars, the mean ± standard deviation.

To detect the apoptosis of GBM cells were affected by HUC-MSCs supernatants, GBM cells U251, U87-MG, LN-428, and RG-2 were treated with HUC-MSCs supernatants for 48 h and the changes in apoptosis of GBM cells were detected by TUNEL assay. TUNEL assay is used to detect DNA breaks formed during the final phase of apoptosis when DNA fragmentation takes place. It is important to validate the induction of cell apoptosis by using DNA-fragmentation assays. From our results, we could find that the cells in the experimental group frequently underwent apoptosis compared to the control group. The apoptosis of RG-2 was the most pronounced, followed by U251 and U87-MG (Fig. 3A). LN-428 apoptosis was less pronounced than that of RG-2, U251, and U87-MG (Fig. 3A). To further verify that GBM cell apoptosis was affected by HUC-MSCs supernatants, we examined the treatment of GBM cells with HUC-MSCs supernatants for 48 h by flow cytometry. The results suggested that after GBM cells were treated with HUC-MSCs supernatants, the percentages of early, late and total apoptotic cells in the RG-2 cells were 24.60% (4.75% in control), 6.76% (1.22% in control) and 31.36% (5.97% in control), respectively. The percentages of early, late, and total apoptotic cells in U251 cells were 14.2% (4.53% in control), 2.44% (1.15% in control), and 16.64% (5.68% in control), respectively. The percentages of early, late, and total apoptotic cells in U87-MG cells were 17.5% (4.93% in control), 2.53% (1.09% in control), and 20.03% (6.02% in control), respectively. The percentages of early, late, and total apoptotic cells in LN-428 cells were 5.20% (1.88% in control), 3.35% (1.62% in control), and 8.55% (3.50% in control) respectively (Fig. 3B). From the above results, it can be seen that HUC-MSCs supernatants promoted apoptosis of GBM cells compared to the control, especially RG-2 (*P* < 0.001) and U87-MG (*P* < 0.001). U251 (*P* < 0.01) was slightly weaker than that of RG-2 and U87-MG, and apoptosis in these three cells occurred at an early stage. However, the apoptotic effect of HUC-MSCs supernatants on LN-428 (*P* < 0.05) was significantly poorer (Fig. 3B and E).

We also examined the expression of proteins associated with GBM apoptosis, such as Bcl-2, Caspase 3, Cleaved-Caspase 3, and Caspase 8. Western Blot results suggested that the expression levels of Bcl-2, Caspase 3, Cleaved-Caspase 3, and Caspase 8 were affected by HUC-MSCs supernatants. In RG-2 cells, compared to the control group, the expression levels of the anti-apoptotic protein Bcl-2 were weakened (65% reduction), and the expression levels of the pro-apoptotic proteins Caspase 3 (42% increase), Cleaved-Caspase 3 (39% increase) and Caspase 8 (62% increase) were increased in the treatment group. In U251, U87-MG, and LN-428, the expression levels of the anti-apoptotic protein Bcl-2 were attenuated (35%, 42%, and 42% reduction). The expression levels of pro-apoptotic proteins Caspase 3 (31%, 39%, and 77% increase), Cleaved-Caspase 3 (61%, 55%, 57% increase), and Caspase 8 (31%, 36%, and 62% increase) were increased in the treated group compared to the control group (Fig. 3C). The results of ICC further supported the above inference (Fig. 3D). These data suggested that GBM cell apoptosis was promoted by HUC-MSCs supernatants.

### HUC-MSCs Supernatants Inhibited GBM Cells Migration and Invasion

**Figure 4:**
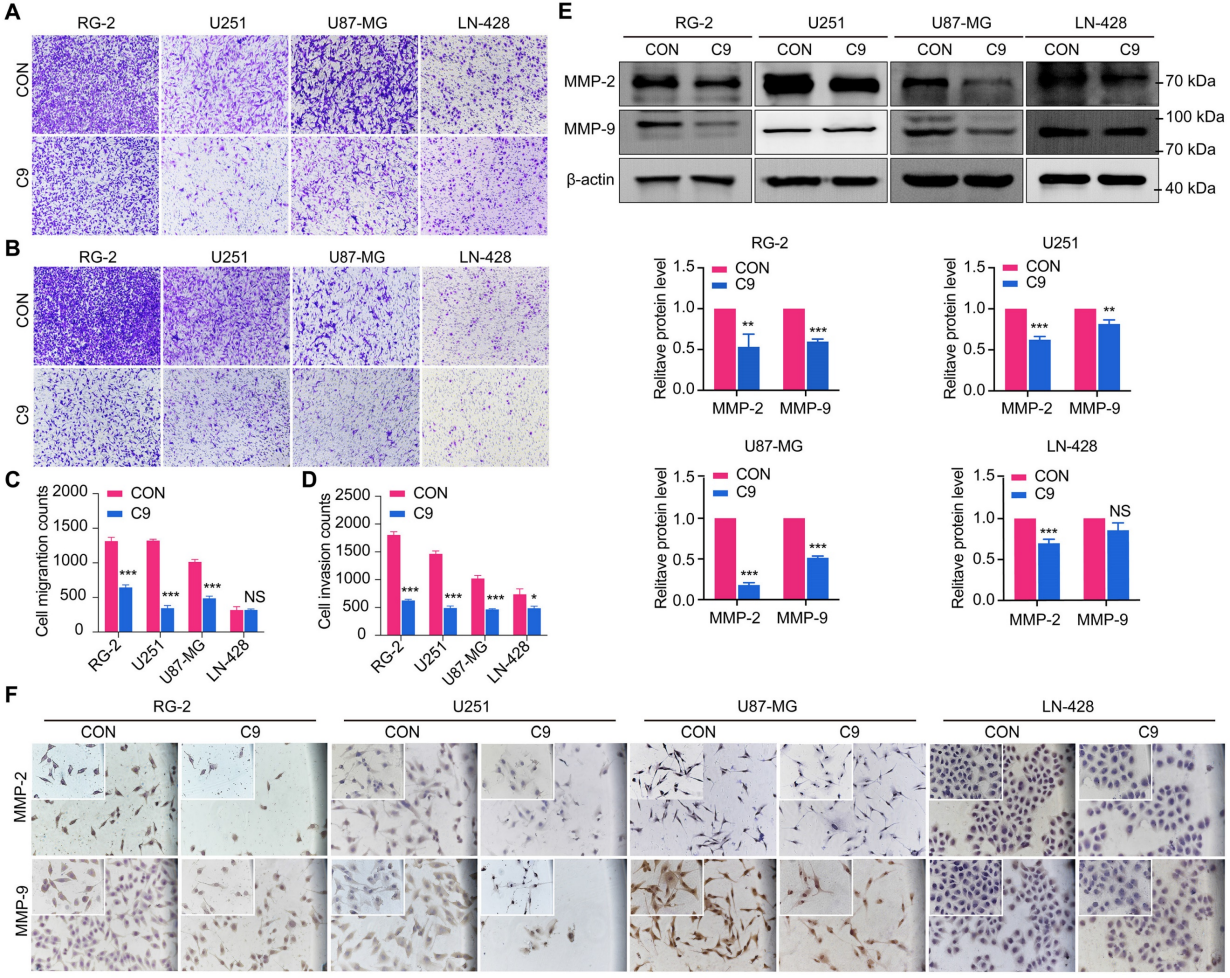
After 48 h treatment with 9 mg/ml HUC-MSCs supernatants, the migration and invasion ability of U251, U87-MG, LN-428, and RG-2 cells were inhibited. **(A)** Transwell Migration assay. **(B)** Transwell Invasion assay. **(C)** Four GBM cell lines were statistically analyzed for migration. **(D)** Four GBM cell lines were statistically analyzed for invasion. **(E)** and **(F)** Changes in the expression of migration-associated proteins MMP-2 and MMP-9 in GBM cells: **(E)** Western Blot. β-actin was used as a qualitative and quantitative control. **(F)** ICC. CON, 0 mg/ml HUC-MSCs supernatants; C9, 9 mg/ml HUC-MSCs supernatants; ICC, Immunocytochemistry. NS, no statistical significance (*P* > 0.05); * with statistical significance (*P < 0.05, ** *P* < 0.01, *** *P* < 0.001 vs CON group). The error bars, the mean ± standard deviation.

Transwell experiments were used to determine whether HUC-MSCs supernatants affect the migration and invasion of GBM cells, we discovered that HUC-MSC supernatants blocked the migration and invasion abilities of GBM cells. The results showed that the migratory ability of GBM cells was significantly affected by HUC-MSCs supernatants in RG-2, U251, and U87-MG cells compared to the control group, especially in RG-2 cells. However, HUC-MSCs supernatants had no effect on LN-428 migratory ability, and quantification revealed no statistical difference *P* > 0.05 (Fig. 4A and C). The Transwell invasion assay results for RG-2, U251 and U87-MG showed the same results as the migration assay. In contrast, the effect of HUC-MSCs supernatants on the invasion ability of LN-428 was existed but weak (Fig. 4B and D). To further confirm that HUC-MSCs supernatants affected GBM cell migration and invasion, we examined the expression of proteins associated with GBM cell migration and invasion, such as MMP-2, MMP-9, and VEGFA. MMP-2, MMP-9, and VEGFA expression were reduced in RG-2, U251, U87-MG, and LN-428 after 48 h of treatment with 9 mg/ml HUC-MSCs supernatants, according to Western blot results. That is, the expressions of MMP-2 (82%), MMP-9 (52%), and VEGFA (44%) were most significantly diminished in U87-MG cells. The migratory and invasive abilities of RG-2 and U251 were weaker than those of U87-MG, with MMP-2 expressions reduced by 47% and 37%, respectively, in RG-2 and U251 cells, and MMP-9 expressions by 40% and 20%, respectively. VEGFA expression was reduced by 30% and 40% in RG-2 and U251 cells, respectively. The expressions of MMP-2 (30%), MMP-9 (13%), and VEGFA (37%) were the worst in LN-428 cells (Fig. 4E and Fig. 6B). The results of ICC further supported the above inference (Fig. 4F and Fig. 6A). In summary, the migratory and invasive ability of GBM cells was inhibited by HUC-MSCs supernatants.

### HUC-MSCs Supernatants Inhibited GBM Cell Growth through Down-regulation of IL-6/JAK2/STAT3 Expression

The IL-6/JAK2/STAT3 signaling pathway plays a critical role in the progression of glioma 27, 37. As a result, we investigated whether HUC-MSCs supernatants could influence the function of the IL-6/JAK2/STAT3 signaling pathway in GBM cells. We extracted total protein from HUC-MSCs supernatant-sensitive RG-2, U87-MG, and LN-428 cells without and with 9 mg/ml HUC-MSCs supernatants treatment for ICC and Western Blot analysis. Consistent with these clinical findings 27, our findings confirmed an increase in IL-6 and JAK2 expression in GBM cells RG-2, U251, U87-MG, and LN-428.

**Figure 5:**
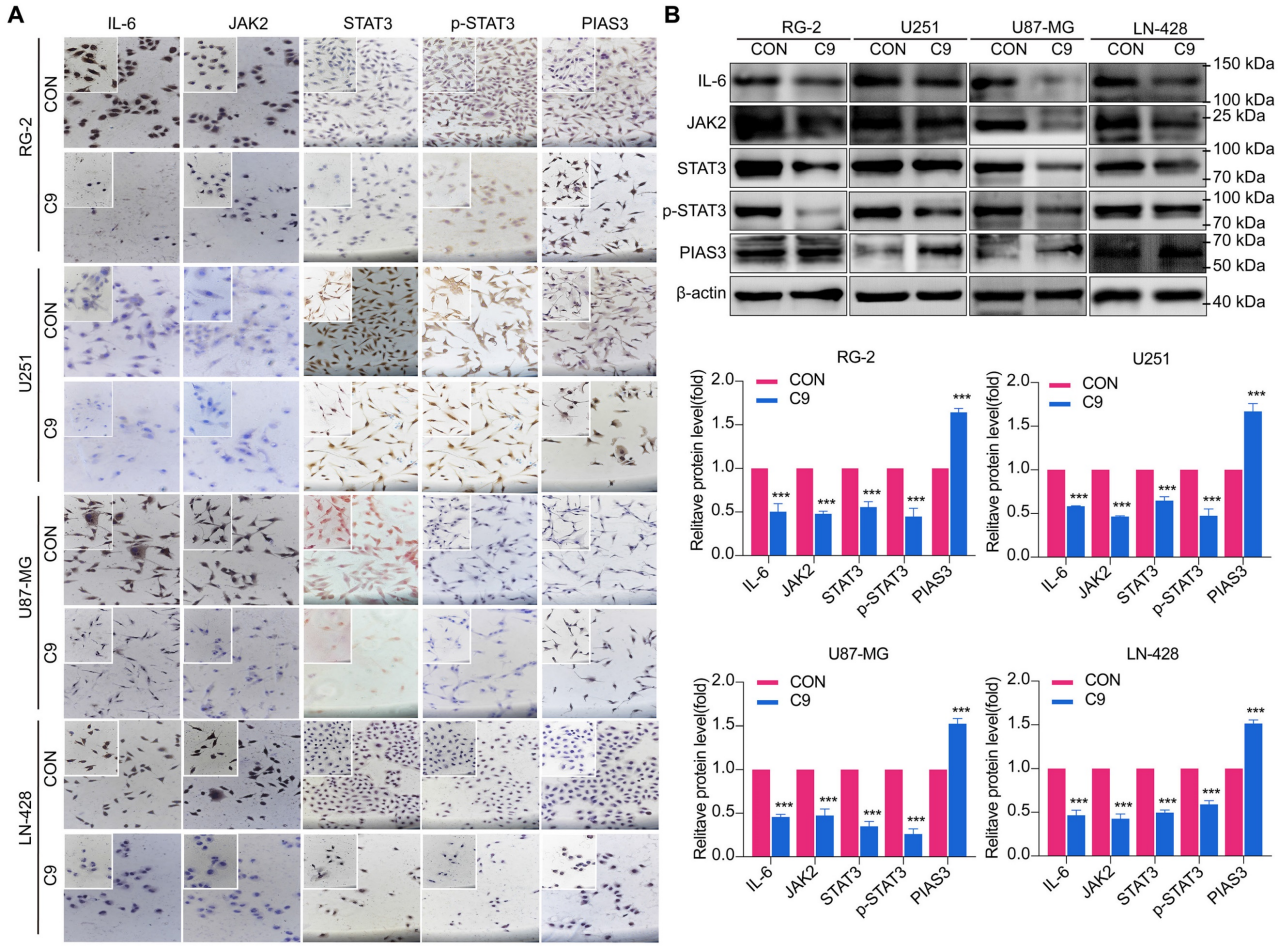
Changes in STAT3 signaling pathway in U251, U87-MG, LN-428, and RG-2 cells after 48 h treatment without (CON) or with (C9) HUC-MSCs supernatants. **(A)** ICC examination (× 20 and × 40), and **(B)** Western Blot analyses of IL-6, JAK2, STAT3, p-STAT3, and PIAS3. β-actin was used as a qualitative and quantitative control. CON, 0 mg/ml HUC-MSCs supernatants; C9, 9 mg/ml HUC-MSCs supernatants. *P < 0.05, **P < 0.01 and ***P < 0.001 vs CON group; the error bars, the mean ± standard deviation.

However, the expression levels of IL-6 and JAK2 decreased after HUC-MSCs supernatants treatment for 48 h (Fig. 5A and B). From Western Blot results, we showed that U87-MG cells were treated with 9 mg/ml HUC-MSCs supernatants for 48 h, IL-6 expression was reduced by 54% compared to the control, and its expression was also decreased by different levels in RG-2 (50%), U251 (42%) and LN-428 (52%) cells, respectively. JAK2 expression was reduced in U87-MG (54%), RG-2 (52%), U251 (53%), and LN-428 (48%) cells (Fig. 5A and B). The results of ICC showed that STAT3 was expressed in all four normal cultured GBM cell lines with significant nuclear translocation, but after treatment with 9 mg/ml HUC-MSCs supernatants, the expression of STAT3 was down-regulated in the four GBM cell lines and nuclear translocation was also reduced (Fig. 5A). The results of Western Blot showed that after GBM cells were treated with 9 mg/ml HUC-MSCs supernatants for 48 h, compared with the control group, the ratio of p-STAT3/STAT3 in RG-2, U251, and U87-MG cells was reduced by 33%, 35%, and 48%, respectively. This indicated that activated STAT3 was reduced in RG-2, U251, and U87-MG cells, especially in U87-MG cells. However, the expression level of p-STAT3 was higher than that of STAT3 in LN-428, which indicated that LN-428 was least inhibited by HUC-MSCs supernatant. Activated STAT3 is expressed at the p-STAT3 level. PIAS3 was lowly expressed in normally treated cells 30, whereas the expression of PIAS3 was elevated by 52% after 9 mg/ml HUC-MSCs supernatant treatment for 48 h. Phosphorylation of STAT3 was also inhibited in U251 (67%) and RG-2 (55%), and LN-428 (41%) cells after 48 h treatment with 9 mg/ml HUC-MSCs supernatants compared to the control, while PIAS3 was persistently highly expressed in U251 (79%) and RG-2 (64%) and LN-428 (51%) (Fig 5B). In conclusion, the growth inhibition of GBM cells may be mediated by the STAT3 signaling pathway.

### Growth Factor Reduction of STAT3 Signaling Pathway was Restrained by HUC-MSCs Supernatants

**Figure 6:**
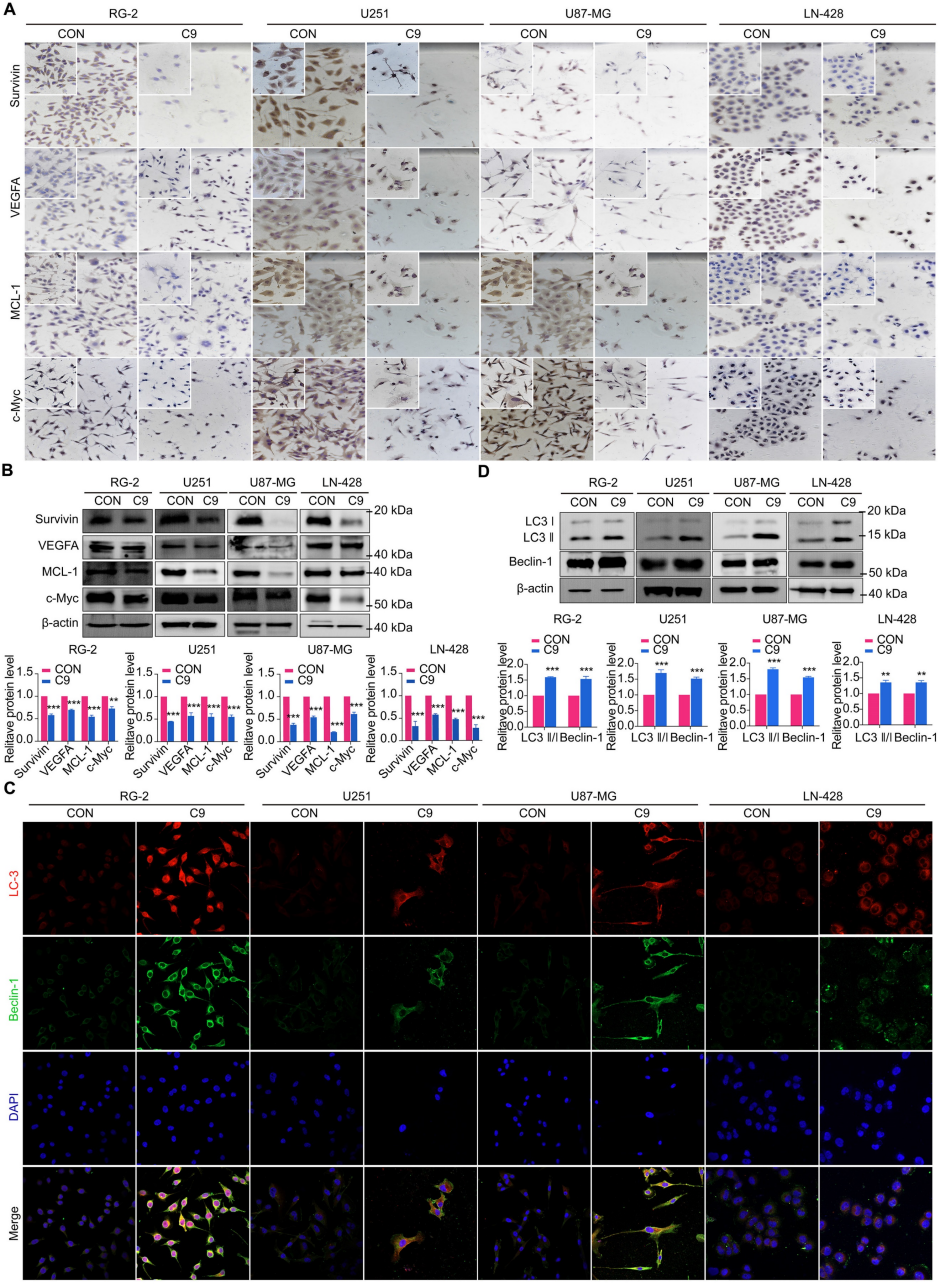
Examinations of MCL-1, Survivin, VEGFA, c-Myc, LC3 ӀӀ/Ӏ and Beclin-1 expression in U251, U87-MG, LN-428, and RG-2 cells without (CON) and with (C9) HUC-MSCs supernatants treatments for 48 h. **(A)** ICC examination (× 20 and × 40) and **(B)** Western blot analyses of MCL-1, Survivin, VEGFA, and c-Myc. β-actin was used as a qualitative and quantitative control. **(C)** IF experiments were performed by laser confocal microscopy (× 20) to examine the expression of the autophagy-related proteins LC3 ӀӀӀ/Ӏ and Beclin-1. and **(D)** Western Blot analyses of LC3 ӀӀ/Ӏ and Beclin-1. β-actin was used as a qualitative and quantitative control. CON, 0 mg/ml HUC-MSCs supernatansts; C9, 9 mg/ml HUC-MSCs supernatants. *P < 0.05, **P < 0.01 and ***P < 0.001 vs CON group; the error bars, the mean ± standard deviation.

STAT3 downstream genes (MCL-1, Bcl-2, Survivin, VEGFA, and c-Myc) play an active role in cell proliferation and maintenance in gliomas 38, 39. Therefore, we analyzed their expression status in four GBM cell lines with or without 9 mg/ml HUC-MSCs supernatants treatment. Western Blot and ICC results showed that MCL-1 levels decreased by 79% in HUC-MSCs supernatants-treated U87-MG cells after 48 h compared to the corresponding controls. HUC-MSCs supernatants-treated MCL-1 decreased by 45% in U251 cells, 45% in RG-2 cells, and 53% in LN-428 cells (Fig. 6A and B). In addition, the results showed that Survivin, VEGFA, and c-Myc levels were lower in U251, RG-2, LN-428, and U87-MG cells than in normal glioma cells. In particular, in U87-MG cells, Survivin, VEGFA, and c-Myc levels were 62%, 44%, and 39%, which were lower than those in normal glioma cells (Fig. 6A and B). In conclusion, the above data suggested that the STAT3 signaling pathway is negatively regulated by HUC-MSCs supernatants, thus enabling improved GBM cells' therapeutic efficacy.

### HUC-MSCs Supernatants Promoted Autophagy Downstream of STAT3 by Inhibiting the Signaling Pathway of STAT3

Whether HUC-MSCs supernatants effects autophagy in GBM cells through the STAT3 signaling pathway. We examined the proteins of RG-2, U251, U87-MG, and LN-428 cells after 48 h treatment with HUC-MSCs supernatants by IF assay. The autophagy-related proteins LC3 ӀӀ/Ӏ and Beclin-1 were found to accumulate in RG-2, U251, U87-MG, and LN-428 cells (Fig. 6C). The results of the Western Blot analysis were consistent with the IF results. After treatment with 9 mg/ml HUC-MSCs for 48 h, the results suggested that the expressions of LC3 ӀӀ/Ӏ, Beclin-1 were most significantly elevated in U87-MG cells by 80% and 55%. LC3 ӀӀ/Ӏ, Beclin-1 was slightly less expressed in U251 and LN-428 cells. At the same time, expression levels were lowest in LN-428 cells at 35% and 32% (Fig. 6D). Taken together, HUC-MSCs supernatants can promote autophagy in GBM cells by negatively regulating the STAT3 signaling pathway.

### HUC-MSCs supernatants inhibit GBM cell proliferation and migration in a STAT3-dependent manner

**Figure 7:**
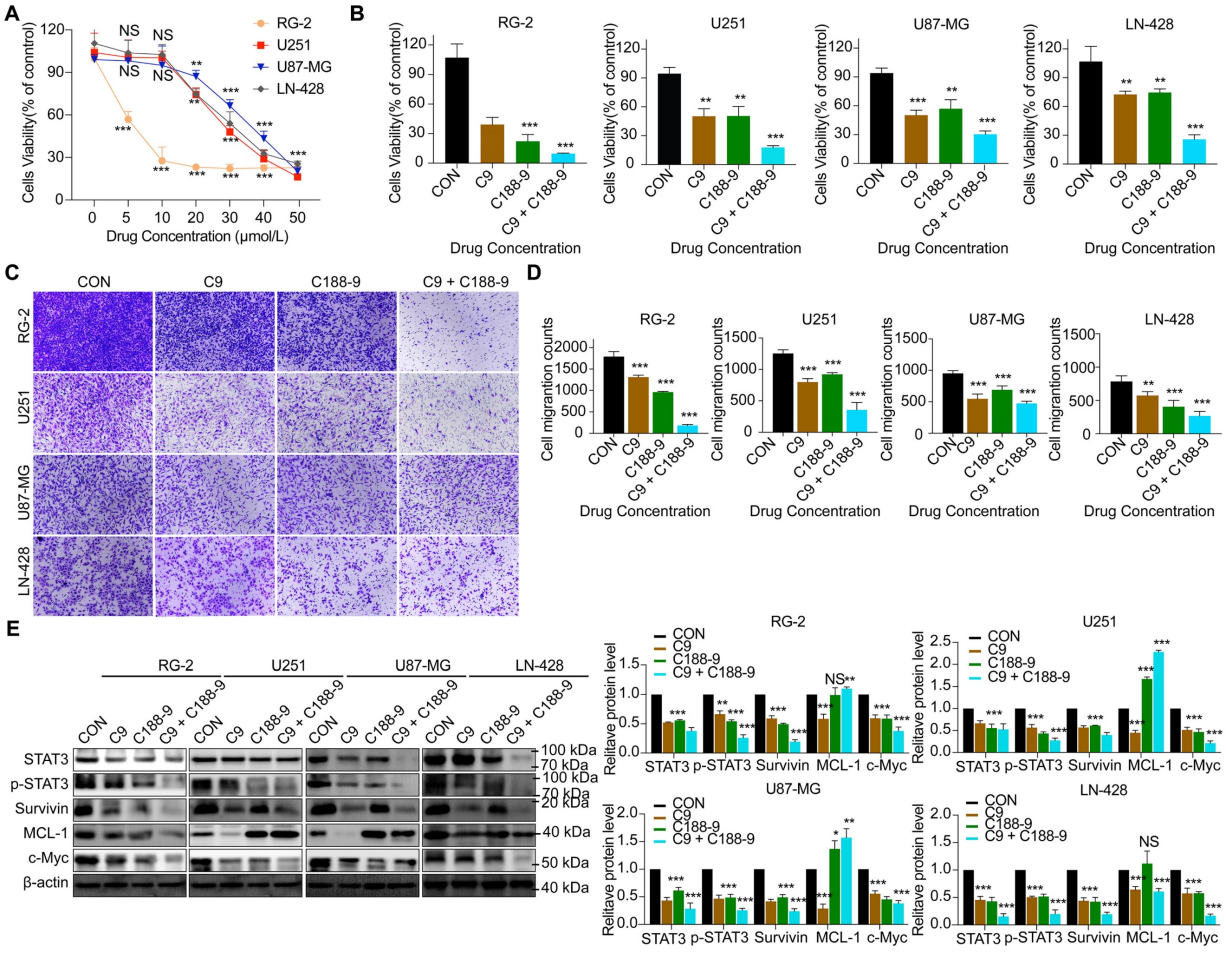
Alterations in STAT3 signaling pathway after 48 h of STAT3 inhibitor C188-9 applied to U251, U87-MG, LN-428, and RG-2 cells.** (A)** CCK-8 assays showing the effect of C188-9 in U251, U87-MG, LN-428, and RG-2 cells. **(B)** CCK-8 assays showing the effect of HUC-MSCs supernatants or/and C188-9 in U251, U87-MG, LN-428, and RG-2 cells. **(C)** Transwell Migration assay. **(D)** Four GBM cell lines were statistically analysed for migration. **(E)** Western Blot analyses of STAT3/p-STAT3 and downstream related proteins Survivin, MCL-1, and c-Myc expression. β-actin was used as a qualitative and quantitative control. CON, Blank control group; C9, 9 mg/ml HUC-MSCs supernatants; C188-9, N‐(1ʹ,2‐Dihydroxy‐1,2ʹ‐binaphthalen‐4ʹ‐yl)‐4‐methoxybenzenesulfonamide; STAT3 inhibitor; *P < 0.05, **P < 0.01 and ***P < 0.001 vs CON group; the error bars, the mean ± standard deviation.

To determine whether HUC-MSCs supernatants inhibit GBM cells' proliferation and migration in a STAT3-dependent manner. C188-9 inhibited the STAT3 gene in GBM cells, so we used HUC-MSCs supernatants to treat them. From the CCK-8 results, we observed that C188-9 was maintained for 48 h, and U251, U87-MG, LN-428, and RG-2 cells showed concentration-dependent. On balance, we chose 20 μmol/L as the effective concentration (Fig. 7A). In addition, from the CCK-8 results, it was observed that although single HUC-MSCs supernatants inhibited the proliferation and migration of U251 (47.4 ± 11.1%) (*P* < 0.01), U87-MG (50.0 ± 5.5%) (*P* < 0.001), LN-428 (28.1 ± 4.3%) (*P* < 0.01), and RG-2 (63.7 ± 13.7%) (*P* < 0.001) cells, the simultaneous action of HUC-MSCs supernatants and C188-8 further inhibited their proliferation and migration (Fig. 7B). That is to say, HUC-MSCs supernatants to treat U251, U87-MG, LN-428, and RG-2 cells, whose STAT3 gene was inhibited by STAT3 inhibitor (C188-9). U251 (82.4 ± 2.8%) (*P* < 0.001), U87-MG (67.6 ± 6.8%) (*P* < 0.001), LN-428 (71.5 ± 9.6%) (*P* < 0.001), and RG-2 (90.3 ± 0.8%) (*P* < 0.001) cells proliferation was further inhibited by HUC-MSCs supernatants (Fig. 7B). Transwell migration assays also showed the same results, especially that HUC-MSCs supernatants or/and C188-9 most significantly inhibited the migration of RG-2 cells (Fig. 7C and D). Western Blot results showed that HUC-MSCs supernatants treat U251, U87-MG, LN-428, and RG-2 cells, whose STAT3 gene was inhibited by STAT3 inhibitor (C188-9). STAT3/p-STAT3 experiments in U251 (53.5%/72.3%), U87-MG (71.6%/74.3%), LN-428 (84.6%/80.3%), and RG-2 (62.3%/74.0%) cells were further inhibited by HUC-MSCs supernatants (Fig. 7E). The expression of STAT3 downstream proteins, Survivin and c-Myc, were also further suppressed in U251 (60.3%/78.0%), U87-MG (77.3%/58.1%), LN-428 (80.6%/83.3%), and RG-2 (80.6%/74.3%) cells. It is worth noting that MCL-1 does not seem to be inhibited by C188-1, and even further promotes the expression of MCL-1. There is no relevant report in the previous literature. We think this may be related to the domain of C188-9 targeting GBM cells, we identified C188-9 as a potent small-molecule that targets the pY peptide-binding site in the SH2 domain of STAT3 34, 35. That is, disruption of the pY peptide-binding site in the SH2 region of STAT3 in GBM cells did not affect MCL-1 expression. In summary, HUC-MSCs supernatants inhibit GBM cell proliferation and migration in a STAT3-dependent manner.

## Discussion

GBM in gliomas is rapidly growing, aggressive, difficult to detect, has a poor prognosis, a high recurrence rate, and is radiotherapy insensitive 40, 41. Given that many anti-GBM drugs, at high doses, cause significant toxicity and BBB obstruction in the brain and/or body 42. The presence of these barriers makes it difficult for many drugs to achieve the desired therapeutic efficacy. As a result, a more effective strategy for treating GBM to supplement or replace existing regimens is urgently needed. HUC-MSCs inhibit tumor growth by secreting relevant cytokines on their own. Studies have shown that HUC-MSCs can induce apoptosis and promote benign progression in oesophageal, ovarian, and leukemia cancer cells 18-20. However, limitations in delivery and risks of instability in humans are both concerns for HUC-MSCs as cell therapy 19, 43. HUC-MSCs supernatants, which contain extracellular vesicles and released bioactive molecules, are a viable cell-free therapy alternative 44. Its activity has the potential to significantly alter neighboring cells' key cellular functions such as survival, apoptosis, maturation, and differentiation 45. Furthermore, it retains the properties of HUC-MSCs, such as the ability to cross the BBB and tumor propensity. Therefore, in our study, we extracted the supernatant of HUC-MSCs for the treatment of GBM cells. We found that HUC-MSCs supernatants can be purified and freeze-dried into a lyophilized powder for large-scale production, easier storage, and long-term stability of biological activity.

**Figure 8:**
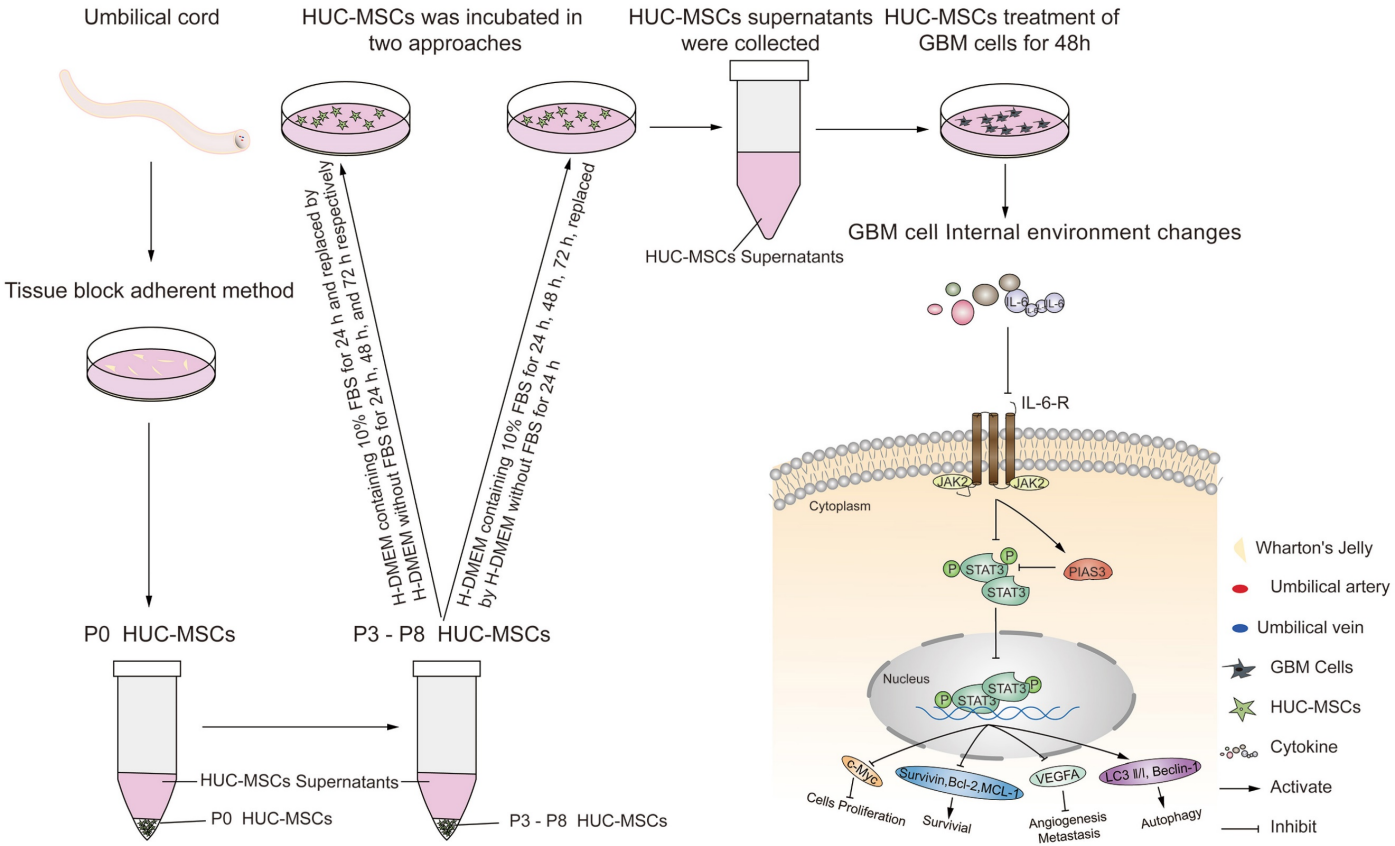
Schematic diagram.

Although previous research has suggested that the immunosuppressive and antitumor effects induced by MSCs on tumor cellular metabolism may be the result of direct cell-cell interactions 46, 47, there has been little investigation into extracting the supernatant of HUC-MSCs at different times to resist GBM. Our study showed that the HUC-MSCs supernatant was effective and feasible for tumor suppression of GBM cells. We found that the HUC-MSCs supernatants inhibited the proliferation of GBM cells to varying degrees. It is unidentified whether the drug concentration of HUC-MSCs supernatants is hindered by incubation time and FBS. To address these issues, we used two approaches to collect HUC-MSCs supernatants (Fig. 8) and incubated GBM cells at various concentrations and times (Table 1). According to the CCK-8 results, HUC-MSCs supernatants inhibited the growth of GBM cells in a concentration-dependent but not time-dependent manner (Fig. 1A and B). Considered collectively, we used H-DMEM containing 10% FBS for 48 h, we then replaced H-DMEM without FBS to continue incubation for 24 h before collecting 9 mg/ml HUC-MSCs supernatants as the effective concentration for subsequent experiments (Fig. 1C). HUC-MSCs supernatants' inhibition of GBM cells could not be separated from changes in GBM cell apoptosis. HUC-MSCs supernatants were found to affect the proliferation and apoptosis of these four GBM cells in multiple experiments. Our data clearly demonstrated that HUC-MSCs supernatants inhibited GBM cell proliferation while also promoting apoptosis. Among them, rat RG-2 cells were the most sensitive, followed by LN-428 and U87-MG, and U251 was the least sensitive. Changes in the cell cycle are also important in the inhibition of GBM cells by HUC-MSC supernatants. Cyclins and cyclin-dependent protein kinases (CDKs) have been shown in studies to play an important regulatory role in cell cycle progression 48. Cyclin A2, CDK2, Cyclin D1, and CDK4 are essential to cell cycle regulatory proteins. Cyclin A2 and CDK2 were significantly downregulated in RG-2, U251, and U87-MG cells by HUC-MSC supernatants, whereas Cyclin D1 and CDK4 were downregulated in LN-428 cells (Fig. 2B). Given that MSCs can gravitate to tumor sites and inhibit tumor cell growth 49. With Transwell assays, we found that HUC-MSCs could converge towards GBM cells *in vitro*, providing a robust scientific foundation for *in vivo* experiments on HUC-MSCs targeting GBM (Fig. 2C).

To explore the molecular mechanisms influenced by HUC-MSCs supernatant, we glanced into the role of the IL-6/JAK2/STAT3 signaling pathway in the inhibition of GBM cell growth by HUC-MSCs supernatants. STAT3 proteins suppress antitumor immunity and accelerate tumor progression. STAT3 activation has been observed in many human cancers, including breast, melanoma, and thyroid cancer 50, 51. Evidence points to STAT3 playing a critical role in GBM development and progression 52, and its transcriptional activation is regulated by IL-6-mediated JAK2 27. STAT3 is an oncogenic transcription factor whose structural and persistent activation of its proteins is highly regulated. STAT3 bound to the promoters of target genes in the nucleus, inducing transcription of genes for cell proliferation (such as cyclin D1, CDK2, and c-Myc), survival (such as MCL-1, Bcl-2, and survivin), angiogenesis-promoting (such as VEGFA) and invasiveness and/or metastasis (such as MMPs) (Fig. 8) 31, 53. STAT3 signaling has been linked to apoptosis and autophagy in cancer cells 30, 51. For this reason, it was speculated that the biological consequences of STAT3 inactivation could be mediated by HUC-MSCs supernatants. Using Western Blot and ICC analysis, this study revealed that HUC-MSCs supernatants suppressed the expression of STAT3-regulated proteins IL-6 and JAK2, thereby downregulating STAT3 and p-STAT3 expressions in GBM cells (Fig. 5). Furthermore, the expressions of STAT3 signaling pathway downstream of cellular proliferation-related proteins c-Myc, survival-related proteins MCL-1, Bcl-2 and survivin, and angiogenesis-promoting proteins VEGFA were also observed. From our experimental results, the expression of Survivin, MCL-1, Bcl-2, VEGFA, and c-Myc in GBM cells has been down-regulated in the presence of HUC-MSCs supernatants (Fig. 6A and B). These observations suggested that the IL-6/JAK2/STAT3 signaling pathway in GBM cells was blocked by HUC-MSCs supernatants. To validate our findings, we used C188-9 to inhibit STAT3 gene expression and found that HUC-MSCs supernatants inhibit the proliferation and migration of GBM cells in a STAT3-dependent manner (Fig. 7E). This finding raises the possibility that HUC-MSCs supernatants is responsible for inhibiting the IL-6/JAK2/STAT3 signaling pathway in GBM cells.

In order to inhibit STAT3 signaling in GBM cells. Subsequently, we sought inhibitors upstream of STAT3, PIAS3, which is one of the most important inhibitors of the STAT3 signaling pathway 54. PIAS3 blocks STAT3 activity and thus STAT3-mediated activation of downstream genes 54. PIAS3 is upregulated to inactivate STAT3, promoting apoptosis and autophagy in tumor cells. We used Western blot to determine the effect of PIAS3 by HUC-MSCs supernatants in GBM cells. The results showed a positive correlation between PIAS3 and the concentration of HUC-MSCs supernatants (Fig. 5). This provides evidence that PIAS3 plays an important role in regulating the STAT3 signaling pathway in GBM cells. To further confirm the role of PIAS3, we used ICC assays to determine that PIAS3 inhibited the STAT3 signaling pathway in GBM cells pretreated with HUC-MSCs supernatants as described above. The present study showed that PIAS3, an upstream inhibitor of the STAT3 signaling pathway, is one of the mechanisms by which HUC-MSCs supernatants inhibit tumor cells. Thus, HUC-MSCs-induced transactivation of the STAT3 signaling pathway may be a potential therapeutic strategy for GBM cells. It should be noted that the degree of STAT3 signaling pathway inhibition varied in different GBM cells, especially in U87-MG cells that were subs-sensitive to HUC-MSCs supernatants. This may be because HUC-MSCs supernatants are involved in antitumor mechanisms differently in U87-MG cells, and further experiments are needed to investigate these issues more fully.

Autophagy is involved in several pathophysiological regulatory processes in humans 55. So far, no report has been available concerning the status of autophagy and its relevance to HUC-MSCs supernatants' sensitivity in GBM cells either *in vitro* or *in vivo*. Some studies have shown an important relationship between STAT3 signaling in apoptosis and autophagy in tumor cells 19, 54, 56. We examined autophagy signaling downstream of the STAT3 signaling pathway, such as Beclin-1 and LC3 protein expressions, in GBM cells by Western Blot combined with laser confocal microscopy. The results suggested that Beclin-1 expression was upregulated by HUC-MSCs supernatants and LC3 Ӏ was converted to LC3 ӀӀ in response to HUC-MSCs supernatants in GBM cells (Fig. 6C and D). Thus, the STAT3 signaling pathway promoted autophagy and apoptosis in GBM cells to enhance the therapeutic effect of GBM cells.

Taken together, the results of this study highlight that although we understand how HUC-MSCs supernatant and GBM cells interact, there are still many questions and obstacles on how this could be used to develop targeted therapies for GBM cells.

## Conclusion

HUC-MSCs supernatants had a pernicious effect on GBM. It inhibited the proliferation, migration, and invasion of GBM cells, promoting cell apoptosis and negatively regulating the IL-6/JAK2/STAT3 signaling pathway, thereby improving the therapeutic efficacy of GBM.

## Figures and Tables

**Table 1:** Grouping of glioma cells treated with HUC-MSC culture supernatant.

Cells Lines	Group	HUC-MSCs Supernatant Extract Concentration (mg/ml)	Incubation Time (hours)
RG-2	0-24	0	24
	0-48		48
	0-72		72
	1-24	1	24
	1-48		48
	1-72		72
	3-24	3	24
	3-48		48
	3-72		72
	6-24	6	24
	6-48		48
	6-72		72
	9-24	9	24
	9-48		48
	9-72		72
U251	0-24	0	24
	0-48		48
	0-72		72
	1-24	1	24
	1-48		48
	1-72		72
	3-24	3	24
	3-48		48
	3-72		72
	6-24	6	24
	6-48		48
	6-72		72
	9-24	9	24
	9-48		48
	9-72		72
U87-MG	0-24	0	24
	0-48		48
	0-72		72
	1-24	1	24
	1-48		48
	1-72		72
	3-24	3	24
	3-48		48
	3-72		72
	6-24	6	24
	6-48		48
	6-72		72
	9-24	9	24
	9-48		48
	9-72		72
LN-428	0-24	0	24
	0-48		48
	0-72		72
	1-24	1	24
	1-48		48
	1-72		72
	3-24	3	24
	3-48		48
	3-72		72
	6-24	6	24
	6-48		48
	6-72		72
	9-24	9	24
	9-48		48
	9-72		72

## Abbreviations

GBM: glioblastoma
HUC-MSCs: human umbilical cord mesenchymal stem cells
GSCs: glioblastoma stem cells
CNS: the central nervous system
LGG: low-grade gliomas
HGG: high-grade gliomas
BBB: blood-brain barrier
TMZ: temozolomide
pY: phosphotyrosine
SH: Src-homology
STAT: Signal transducer and activator of transcription
PIAS3: Protein inhibitor of activated STAT3
TUNEL: Terminal Deoxynucleotidyl Transferase dUTP Nick End Labeling
PI: propidium iodide
ICC: Immunocytochemical Staining
IF: Triple Immunofluorescence Staining
SDS-PAGE: sodium doecylulfate--polyacrylamide gel electrophoresis
SD: standard deviation
CDKs: cyclin-dependent protein kinases
MMPs: matrix metalloproteinases
