# Inhibition of STAT3 signaling as critical molecular event in HUC-MSCs suppressed Glioblastoma Cells: Erratum

**DOI:** 10.7150/jca.105504

**Published:** 2024-11-02

**Authors:** Mingming Wang, Yufu Zhang, Min Liu, Yuna Jia, Jing He, Xiangrong Xu, Haiyan Shi, Yunqing Zhang, Jing Zhang, Yusi Liu

**Affiliations:** 1 Department of Cell Biology and Genetics, Medical College of Yan'an University, Yan'an 716000, Shaanxi Province, China; 2 Department of Hepatobiliary Surgery, Affiliated Hospital of Yan'an University, Yan'an 716000, Shaanxi Province, China; 3 Department of Pathology, Affiliated Hospital of Yan'an University, Yan'an 716000, Shaanxi Province, China; 4 Laboratory of Obstetrics and Gynecology, Affiliated Hospital of Yan'an University, Yan'an, 716000 Shaanxi Province, China

In the original version of our article, there was an error in Figure 5A, 6A, 7E. Specifically, the Immunocytochemical Staining (ICC) for U251 STAT3, P-STAT3 C9, VEGFA C9 and U87-MG MCL-1 was used wrong in Figure 5A and Figure 6A, so we replaced with the correct band, and the western blot band for RG-2 actin was used wrong in Figure 7E, so we replaced with the correct band. The correct image is provided below. This correction will not affect the results and conclusions. We are deeply sorry and sincerely apologize for the error and for any inconvenience that may cause to the readers and the editors of this journal.

## Figures and Tables

**Figure 5:**
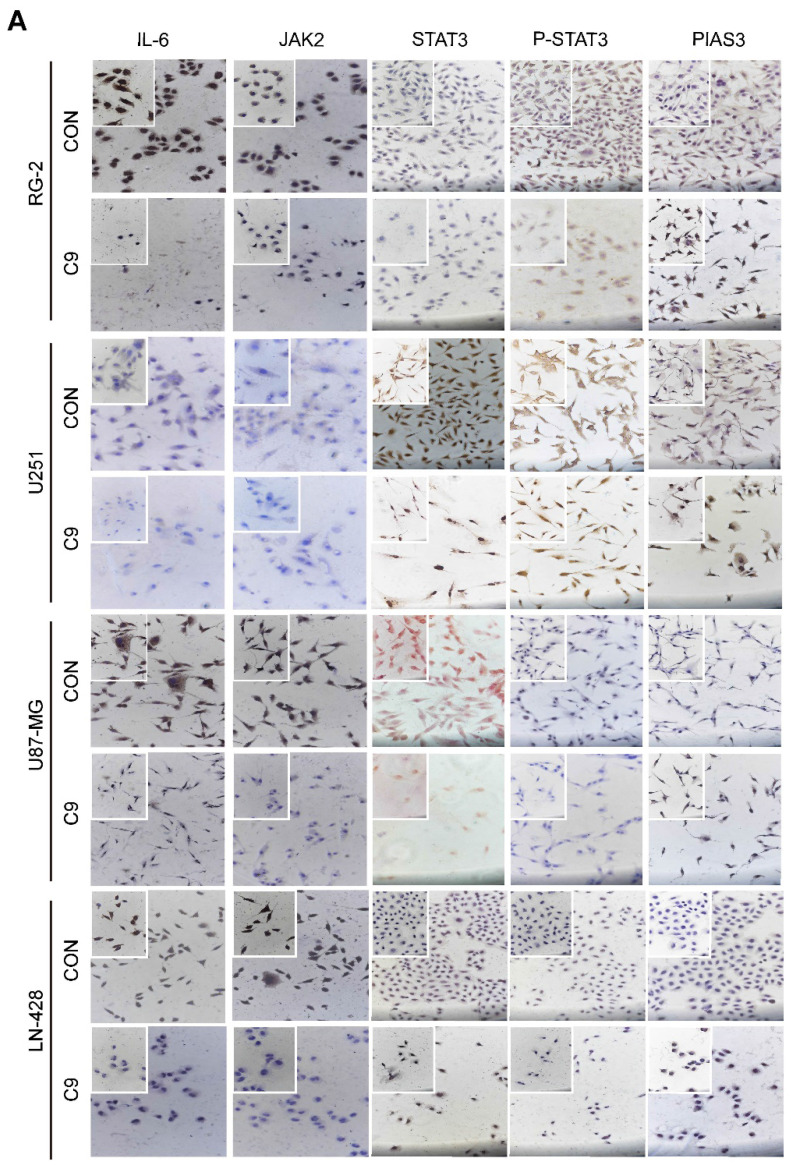
Changes in STAT3 signaling pathway in U251, U87-MG, LN-428, and RG-2 cells after 48 h treatment without (CON) or with (C9) HUC-MSCs supernatants. **(A)** ICC examination (× 20 and × 40), and **(B)** Western Blot analyses of IL-6, JAK2, STAT3, p-STAT3, and PIAS3. β-actin was used as a qualitative and quantitative control. CON, 0 mg/ml HUC-MSCs supernatants; C9, 9 mg/ml HUC-MSCs supernatants. *P < 0.05, **P < 0.01 and ***P < 0.001 vs CON group; the error bars, the mean ± standard deviation.

**Figure 6:**
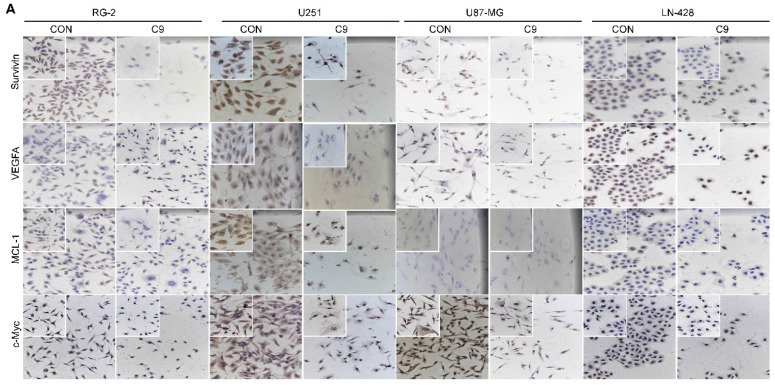
Examinations of MCL-1, Survivin, VEGFA, c-Myc, LC3 ӀӀ/Ӏ and Beclin-1 expression in U251, U87-MG, LN-428, and RG-2 cells without (CON) and with (C9) HUC-MSCs supernatants treatments for 48 h. **(A)** ICC examination (× 20 and × 40) and **(B)** Western blot analyses of MCL-1, Survivin, VEGFA, and c-Myc. β-actin was used as a qualitative and quantitative control. **(C)** IF experiments were performed by laser confocal microscopy (× 20) to examine the expression of the autophagy-related proteins LC3 ӀӀӀ/Ӏ and Beclin-1. and **(D)** Western Blot analyses of LC3 ӀӀ/Ӏ and Beclin-1. β-actin was used as a qualitative and quantitative control. CON, 0 mg/ml HUC-MSCs supernatansts; C9, 9 mg/ml HUC-MSCs supernatants. *P < 0.05, **P < 0.01 and ***P < 0.001 vs CON group; the error bars, the mean ± standard deviation.

**Figure 7:**
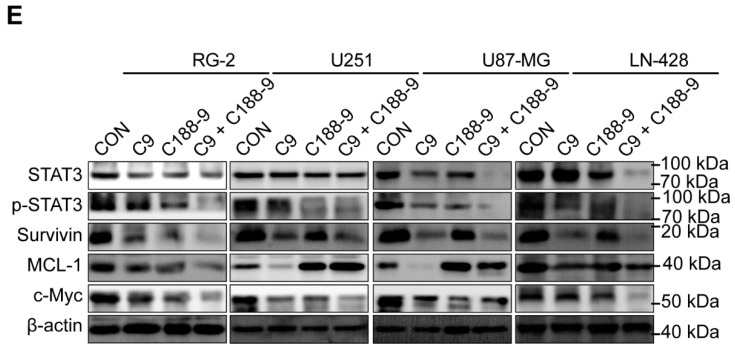
Alterations in STAT3 signaling pathway after 48h of STAT3 inhibitor C188-9 applied to U251, U87-MG, LN-428 and RG-2 cells.** (A)** CCK-8 assays showing the effect of C188-9 in U251, U87-MG, LN-428 and RG-2 cells. **(B)** CCK-8 assays showing the effect of HUC-MSCs supernatant or/and C188-9 in U251, U87-MG, LN-428 and RG-2 cells. **(C)** Transwell Migration assay. **(D)** Four GBM cell lines were statistically analysed for migration. **(E)** Western blot analyses of STAT3/p-STAT3 and downstream related proteins Survivin, MCL-1 and c-Myc expression. β-actin was used as a qualitative and quantitative control. CON, Blank control group; C9, 9 mg/ml HUC-MSCs supernatant; C188-9: N-(1ʹ,2-Dihydroxy-1,2ʹ-binaphthalen-4ʹ-yl)-4-methoxybenzenesulfonamide, STAT3 inhibitor; *P < 0.05, **P < 0.01 and ***P < 0.001 vs CON group; the error bars, the mean ± standard deviation.

